# A randomized controlled trial studying the effectiveness of group medical appointments on self-efficacy and adherence in sickle cell disease (TEAM study): study protocol

**DOI:** 10.1186/s12878-016-0058-4

**Published:** 2016-08-04

**Authors:** Marlous J. Madderom, Jessica Heijdra, Elisabeth M. W. J. Utens, Suzanne Polinder, Anita W. Rijneveld, Marjon H. Cnossen

**Affiliations:** 1 Department of Pediatric Hematology, Erasmus University Medical Center - Sophia Children’s Hospital, Wytemaweg 80, PO Box 2060, 3000 CB Rotterdam, The Netherlands; 2 Department of Child and Adolescent Psychiatry/Psychology, Erasmus University Medical Center – Sophia Children’s Hospital, Wytemaweg 80, PO Box 2060, 3000 CB Rotterdam, The Netherlands; 3 Department of Public Health, Erasmus University Medical Center, ‘s-Gravendijkwal 2030, PO 2040, 3000 CA Rotterdam, The Netherlands; 4 Department of Hematology, Erasmus University Medical Center, ‘s-Gravendijkwal 230, PO Box 2040, 3000 CA Rotterdam, The Netherlands

**Keywords:** Randomized controlled trial, Sickle cell disease, Self-efficacy, Adherence, Group medical appointment, Cost-effectiveness

## Abstract

**Background:**

Sickle cell disease (SCD) is endemic in non-Western countries. Due to migration, the prevalence of SCD in the Netherlands has increased. Adherence to medical treatment is recognized as a major problem area. Therefore, new effective interventions to increase adherence are urgently needed.

**Methods/design:**

The TEAM study is an ongoing randomized controlled trial (RCT) to compare protocolized individual medical appointments (IMA’s; care-as-usual) with protocolized group medical appointments (GMA’s; novel intervention) in pediatric (*n* = 40) and adult (*n* = 60) patients. The study aims to assess the effectiveness of GMA’s (over a three year period) on patients’ self-efficacy, adherence, quality of life, morbidity, hospital admissions and satisfaction with the treating professional; as well as to test the cost-effectiveness of GMA’s. In both the IMA and GMA groups structured assessments will be performed at baseline (start of the study), after 1.5 and after 3 years.

**Discussion:**

This is the first RCT to investigate the effectiveness of GMA’s on self-efficacy and adherence in pediatric and adult patients with SCD, including a cost-effectiveness analysis.

**Trial registration:**

NTR4750 (NL42182.000.12). Registered 13 August 2014.

## Background

### Sickle cell disease and important aspects of care

Sickle cell disease (SCD) is an autosomal recessively inherited red cell disorder caused by an abnormal production of hemoglobin. It is characterized by severe chronic anemia, fulminant infections due to functional asplenia, and repetitive painful vaso-occlusive ischemic “sickle cell crises”. The latter are often provoked by infection, fever, pain, dehydration, cold and stress. Ultimately, multiorgan failure develops, accumulating in avascular necrosis of bones, retinopathy, stroke, cardiomyopathy, pulmonary hypertension and kidney failure, among others. Life expectancy is set at 45 years of age. Causes of death are multifactorial but often due to organ failure caused by the disease. Overall, the direct and indirect costs of SCD treatment are substantial [1].

SCD is endemic in Africa, the countries bordering the Mediterranean Sea (Morocco, Turkey), the Middle East (Iran, Irak) and the Caribbean region (Surinam, Antilles, Haiti and Dominican Republic). Moreover, carriership is 1:7 individuals from these regions. As more than 11 % of the current Dutch population and more than 50 % of the younger population in the larger cities originates from these non-Western countries, neonatal screening for SCD was initiated by the Dutch government on January 1st 2007 [2], aiming to lower age at presentation and to improve timely and adequate patient support. Since neonatal screening was initiated, approximately 60 children with SCD and 800 SCD carriers are born annually [3].

Therapeutic interventions for SCD include: infection prevention by prophylactic antibiotics in the younger population, vaccinations, folic acid supplemention, blood transfusions in combination with iron chelation therapy, hydroxycarbamide and antihypertension treatment. An important part of treatment regimens consists of extensive patient education on the importance of: antibiotic prophylaxis, recognition of provoking risk factors for painful sickle cell crises, early disease symptoms and complications, lifestyle modifications as well as the inheritance of the disease.

Delivering adequate medical care for SCD patients is often difficult due to the fact that most patients present themselves when disease symptoms have already progressed and exhibit poor adherence to medication and medical visits, due to diverse patient related factors which are not easily influenced [4, 5]. An important factor of treatment, and by consequence prognosis, is “self-efficacy”. Self-efficacy is defined as confidence in one’s own capabilities to manage illness. Importantly, it is modifiable as shown by various studies [4, 6–8]. An innovative form of outpatient contact to improve self-efficacy is a protocolized Group Medical Appointment (GMA), in which a protocolized Individual Medical Appointment (IMA; care-as-usual) is incorporated within a group consultation, in the presence of fellow patients and other medical professionals [9, 10] (Table 1).

**Table 1:** Characteristics of the individual- and group medical appointments

	Group medical appointment	Individual medical appointment
Number of patients	6–8	1
Duration of appointment	90 min total	15 min per professional
Severity of disease	Various severities of disease	One severity of disease
Professionals	Treating physician, nurse, clinical geneticist, social worker	Treating physician, nurse, clinical geneticist, social worker
Clinical examination	Behind a screen in conference room or in another room (before or after the appointment)	In physician’s office
Privacy	Confidentiality protected by the group	Completely

We aim to analyze the effectiveness of GMA versus IMA, in pediatric and adult patients with SCD, in a study with a randomized controlled trial (RCT) design.

### Hypotheses

We hypothesize that: 1) GMA will improve self-efficacy, adherence, quality of life and the patient-professional relationship in SCD patients as compared to IMA; 2) and that complementary periodic GMA’s (four in three years) will be a cost-effective approach to intensify support for these patients, since SCD requires time consuming support by treating professionals.

## Methods/Design

In this RCT with a parallel design, effects of protocolized GMA versus protocolized IMA (care-as-usual) will be tested in SCD patients, over a three year period, for children and adults separately.

### Participants

**Fig. 1:**
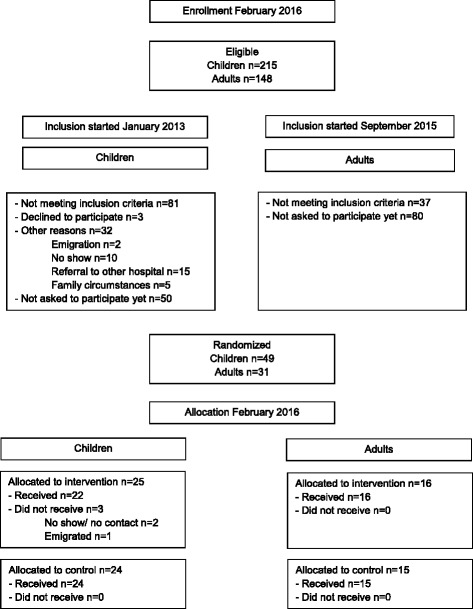
Flowchart

Although the natural course of the disease in our patient population differs according to disease severity, the pathophysiology of the disease, alarming signals, lifestyle alterations and mode of inheritance do not differ. The content of the GMA’s are applicable to our entire patient population and therefore all patients with homozygous or compound heterozygous SCD are eligible. We will exclude patients with a first visit to the outpatient clinic, patients who cannot communicate adequately due to language difficulties and/or hearing problems or patients who have behavioral problems which will limit group functioning (see Fig. 1).

The expected study population will consist of: 40 children and 60 adults with all types of SCD (HbSS, HbSC, HbS beta + and 0 thalassemia) from the Sickle Cell Comprehensive Care Center in the Erasmus Medical Center - Sophia Children’s Hospital Rotterdam, the Netherlands.

All patients/parents will be informed about the study and will be asked to provide permission to use the data for research purposes. Then they will be given one to two weeks consideration time. When the patients/parents agree to participate, written informed consent will be asked by the investigator and the supervising physician.

### Interventions

#### Care-as-usual: IMA

Protocolized care-as-usual consists of an IMA to the (pediatric) hematology department at least every six months with a total of seven IMA’s in a three year period. Monitoring of organ dysfunction is performed by annual laboratory analyses, echocardiography and transcranial Doppler and duplex analysis (1–2 times a year), the latter only in children. Consultation visits are also made to various subspecialists, according to protocol (cardiologist, clinical neurophysiologist, ophthalmologist) and when clinically indicated (pulmonologist, gynecologist, neurologist, nephrologist, anesthesiologist, orthopedic surgeon). All patients included in the study will receive a personal dossier in which sickle cell crises, emergency department visits, hospital admissions and blood transfusions are documented. This is of importance as many patients are concomitantly monitored by physicians at other non-academic hospitals. Furthermore, all patients or parents in our comprehensive care center standardly receive extra reminder letters and are contacted prior to appointments by telephone to keep lost to follow-up to a minimum.

#### Intervention: GMA

In the intervention group, every other outpatient clinic visit will be replaced by a protocolized GMA. GMA was introduced by Noffsinger and Scott and recently promoted by “Kwaliteitsinstituut voor de Gezondheidszorg (CBO)” in the Netherlands, as an effective option to optimize self- efficacy, adherence, as well as treatment regimen and lifestyle alterations in chronically ill patients [9–16]. During a GMA, approximately eight patients and their parents/partners are seen simultaneously by the (pediatric) hematologist, during a 1.5 h session. Since it is important to safeguard the heterogeneity of the groups, we will not invite the same patients (and parents) to each GMA, although sometimes there may be overlap. The protocolized standard of care for our patient population consists of two hospital visits per year. Since GMA’s have been shown to be effective for the motivated patient [12], we have chosen not to exceed the two visits to the hospital. Therefore, during this three year RCT, the protocolized GMA will be performed every other six months, leading to a frequency of once a year, with a total of four GMA’s and three IMA’s in a three year period.

A chairman trained to supervise a GMA, hosts and facilitates the sessions. Patients consent to confidentiality in each session. Although all components of an IMA are incorporated, a GMA allows more time to discuss disease related topics and psycho-education. In addition, information and social support from fellow-patients can greatly improve self-efficacy and quality of life [11, 14] (Table 2). GMA is provided for children and their parents (all ages until approximately 16 years of age) by the pediatric team (a pediatric hematologist, a clinical geneticist, a sickle cell nurse and a social worker) and for teenagers (approximately 16 years and older and in transition to the adult hospital) and adults by the adult team (a hematologist, a clinical geneticist, two sickle cell nurses and a social worker). Economically, GMA appointments are billed as an IMA.

**Table 2:** Structure of (pediatric) sickle cell disease group medical appointment

1. Measurement of weight and height of all participants.
2. Introduction by the appointed chairman with special attention to procedure, privacy and allotted time.
3. Individual interview by treating physician specifically focusing on individual disease symptoms and complications.
4. During the GMA disease-related topics are discussed, in accordance to patient questions or introduced by the chairman:
Disease and therapy
Early identification of sickle cell crises and crises provoking factors
Interventions to influence sickle cell crises provoking factors.
Pain treatment regimens, importance of treating pain early and adequately
Consequences of functional asplenia and importance of anticipating possible consequences (antibiotic prophylaxis, vaccinations, early treatment with broad spectrum antibiotics in case of fever)
Recognition of important or alarming disease symptoms: aggravation of anemia, splenic sequestration, severe sickle cell crises needing hospital attention, infections and respiratory problems, gallstones, symptoms suggestive of cerebral infarctions, cognitive deterioration, priapismus, bone infarctions
Education with regard to pathophysiological effects of treatment options (hydroxycarbamide, chronic blood transfusions)
Importance of healthy lifestyle (enough rest, healthy diet, consciousness of limitations)
Importance of informing school and others of disease
Regular dental consultations and importance of vaccinations according to national guidelines
Lifestyle
Avoidance and early treatment of sickle cell crisis provoking factors (cold, fever/infection, pain, high altitude, dehydration/enough fluids, fatigue and stressful situations) and underlining specific social and physical circumstances with risks: swimming lessons, alcohol, menstrual period, intensive sporting activities
Encouragement of individual self-empowerment, self-efficacy
Frequent contact with Sickle Cell Comprehensive Care Center is encouraged
Support and guidance with regard to job and career choice
Genetics
Explanation of different sickle cell genotypes
Inheritance of the disease and importance of genetic counseling
Explanation of meaning of coinheritance of alpha thalassemia and persistent fetal hemoglobin levels (HbF)
Options with regard to prenatal diagnosis and preimplantation genetic diagnosis
5. At the end of the GMA diagnostic vena punctures and more private individual consultations are performed and, if necessary, physical examinations

#### Previous evidence of GMA-effectiveness

Various studies in adults with other diseases such as diabetes, severe headaches, cardiovascular and urological problems have shown that GMA may enhance patients’ and physician’s satisfaction, patients’ self-efficacy, quality of life, and that it can reduce emergency visits and hospital admissions [12, 14, 17–21]. Moreover, it has been suggested to be cost-effective in chronic diseases requiring extensive patient education to modify disease course and to prevent complications [14, 20, 21].

#### Innovative character

To date, GMA has not been applied in SCD and few have been performed in pediatric populations [22]. We also aim to quantify cost-effectiveness of the intervention, in this time consuming disease, by an economic analysis.

### Outcomes

Primary and secondary endpoints will be measured at baseline (start of the study), after 1.5 years (after two GMA visits) and after 3 years (after four GMA visits), in both groups. Assessments are performed at the hospital, directly before the outpatient visit and in presence of a psychologist.

Primary endpoint:

1. Self-efficacy as measured by the validated Sickle Cell Self- Efficacy Scale [7];

Secondary endpoints:

2. Adherence to prescribed treatment by (pediatric) hematologist, defined by:
  Self-report (Morisky Medication Adherence Scale) [23];
  Variations in adherence (Medication Adherence Report Scale) [24];
  Blood values suggesting adherence to prescribed medication (folic acid, hydroxycarbamide, iron chelation therapy) such as: folic acid (nmol/l), MCV (fl), fetal hemoglobin percentage (HbF %), ferritine (mcg/l);
  Attendance at outpatient clinic visits or imaging examinations (% of total planned).
3. Quality of life as measured with the validated Pediatric Quality of Life Inventory for children [25] and SF-36 for adults [26].
4. Emergency visits and hospital admissions for SCD related symptoms and complications. At *t* = 0, morbidity of preceding 3 years will be measured as baseline. All patients in the study will receive a dossier in which sickle cell crises, emergency department visits, hospital admissions, blood transfusions will be documented.
5. Satisfaction with treating physician and nurse (by visual analogue scale: score 1–10) [27, 28].
6. Measurement of costs and effects in the GMA and IMA group by an economic analysis according to Dutch guidelines and with respect to an increase in self- efficacy.

#### Cost-effectiveness analysis

GMA’s will be compared with IMA’s in accordance with Dutch guidelines [29]. Cost-effectiveness of GMA in SCD will be assessed by calculating the incremental cost-effectiveness ratio (ICER), defined as the difference in costs of GMA compared to care-as-usual (IMA), divided by the average change in effectiveness, with self-efficacy measured with the validated Sickle Cell Efficacy Scale as the primary outcome measure. An economic evaluation will be performed from a healthcare perspective for the period of three years after the start of the intervention. For the calculation of the costs from a healthcare perspective, total medical care costs will be measured (e.g. GMA, inpatient days, professional health caregivers’ activities, medical procedures). Actual medical costs will be calculated by multiplying the volumes of healthcare use with the corresponding unit prices. Data on resource use will be collected from medical files. The cost price of GMA will be determined with the bottom-up micro-costing method [30], which is based on a detailed assessment of all resources used. Therefore, costs for all separate actions and time used by all individual healthcare professionals will be measured. For the calculation of other medical costs, we will use charges as published in Dutch guidelines as a proxy of real costs. We will perform a sensitivity analysis to assess the stability of the results to changes in costs and effectiveness parameters [29].

#### Budget impact analysis

A Budget Impact Analysis (BIA) will be performed in accordance to principles for good BIA practice. A valid framework will be provided according to Markov, leading to insight in budget consequences of desensitization, by a range of predictions based on realistic estimates and scenarios of input parameter values [31]. The likely impact of GMA on center level and also on national health plan budgets.

### Sample size

As the effect size (which varies between Cohen’s *d* = 0.00 and *d* = 0.95) could only be estimated and is best comparable to that shown for cognitive behavioral therapy [32], we presume effect of GMA on endpoints is 0.50 (moderate effect size according to Cohen) [33]. With a power of 0.80 and an alpha of 0.05 (two-tailed), a sample size of 100 patients will suffice (two data sets; children: 40 patients, with 20 in each arm; adults: 60 patients, with 30 in each arm). To adjust for loss to follow up 65 adults and 45 children will be included.

### Randomization

After informed consent, patients (children and adults separately) will be randomized by the investigator with the use of the Trial Online Process registration and randomization program. Allocation will be known to all involved in the study. The randomization process will stratify by severity of disease (with severe SCD defined as: HbSS or HbSβ0 and milder disease defined as: other compound heterozygous forms of SCD). In order to investigate whether the study population is a representative sample of the total population in our hospital we will compare background variables (age, gender, socioeconomic status and diagnosis) between both populations when the study is completed.

## Discussion

This article presents the rationale and design of a RCT to test the effectiveness of GMA versus IMA on self-efficacy, adherence, quality of life and patient-professional relationship in pediatric and adult patients with SCD. Furthermore, effects of GMA on medical costs (e.g., morbidity, hospital admissions) will be tested. This is the first study to investigate the effects of GMA on these parameters in patients with SCD and is the first to evaluate the cost-effectiveness of such an intervention. If the GMA’s prove successful in improving self-efficacy, adherence and cost-effectiveness, this innovation in SCD care may be disseminated at an (inter)national level.

## Trial status

The trial opened to recruitment in January 2013 for the children and in September 2015 for the adults and is still ongoing.

## Abbreviations

BIA, budget impact analysis; CBO, Kwaliteitsinstituut voor de Gezondheidszorg; GMA, group medical appointment; ICER, incremental cost-effectiveness ratio; IMA, individual medical appointment; RCT, randomized controlled trial; SCD, sickle cell disease
